# Patient-reported outcomes after total knee replacement: Questionnaire and functional assessment study

**DOI:** 10.6026/973206300221970

**Published:** 2026-04-30

**Authors:** Avinash Nachkunte, Mustafa Al-Jaafar, Amruta Dashputra

**Affiliations:** 1 Department of Trauma and Orthopaedics, Aneurin Bevan University Health Board, Wales United Kingdom; 2 Department of Pharmacology, NKP Salve Institute Medical Sciences and Research center and Lata Mangeshkar Hospital Nagpur, India

**Keywords:** Total knee replacement (TKR), patient-reported outcomes, quality of life, functional assessment, postoperative recovery, knee function, rehabilitation, joint pain relief, orthopedic surgery, patient satisfaction

## Abstract

Recent studies indicate that although many patients are satisfied with their surgical results after total knee replacement (TKR), there is still concern regarding ongoing levels of pain, limited mobility and differences in the level of satisfaction among patients. Therefore, it is of interest to prospectively evaluate patient-reported outcomes and functional recovery in TKR patients following the completion of at least 6 months post-operative recovery through the application of KOOS, SF-36 and time up, go and 6-minute walk tests. In this study, KOOS Pain scores increased from an average of 45.6 points before surgery to an average of 82.1 points after surgery, while SF-36 physical functioning scores increased from an average of 41.2 points preoperatively to an average of 80.5 points at follow-up (p<0.001). Additionally, functional mobility improved significantly through decreased times on the timed up and go test from 18.4 seconds preoperatively to 10.2 seconds postoperatively (p<0.001) and increased distances walked during the 6-minute walk test from 230.7 meters to 360.8 meters (p<0.001). Furthermore, TKR has led to significant increases in patient satisfaction, functional independence and overall quality of life for TKR patients.

## Background:

To alleviate the chronic pain associated with advanced osteoarthritis, many surgeons today use total knee replacement [1]. However, despite these high surgical success rates, some of these total knee replacement patients have reported having continued problems with pain, etc. after surgery [2]. While clinical outcomes such as pain and radiology can determine how someone has recovered from surgery; there is much evidence to indicate that the patient's perspective of their post-surgical recovery can differ (or not be completely accurate) [3]. In order to have more accurate results and to have better outcomes for patients, it is essential to collect and to analyze the patient's response as well as to collect other objective results (using functional capacity tests) so that it gives a broader view of the patient's recovery/rehabilitation process [4]. Newer research continually proposes and reinforces that the best outcome assessment for any type of surgery is through a patient-centered outcome model; this allows for improved guidance with post-operative care and ultimately improves patient satisfaction [5, 6]. However, post-operative outcomes for total knee replacement surgeries vary considerably between patients based on their demographics and clinical condition; therefore, it is necessary to study these patients in a context-based manner to ensure the best outcomes for them [7]. Therefore, it is of interest to evaluate the outcome measures as determined by the patient as well as their overall quality of life (using questionnaires); to gain further insight as to how successful the patient's surgery has been and if so, how can the patient continue to improve on their overall health and well-being.

## Materials and Methods:

The current study's objective (i.e., a prospective observation) was to evaluate the effectiveness of total knee replacement (TKR) surgery on function health among patients treated with TKR for DJD (degenerative joint disease) in a tertiary-care hospital. A sample of 100 patients (50-80 years of age) was obtained from patients who had undergone TKR over a six-month postoperative period using a consecutive sampling method. Exclusion criteria included any patient with a revision surgery, post-surgery infection or a neurological disorder that interfered with ambulating. Patient outcomes were assessed via the knee injury and osteoarthritis outcome score (KOOS) and the short form health survey 36 (SF-36); patient performance functional assessment was completed using the timed up, go test (TUGT) and the six-minute walk test (6MWT) under controlled conditions by physiotherapists. Age, sex, BMI and how long it has been since surgery were recorded as demographic/clinical variables at time of follow-up. Results from descriptive statistics and paired t-tests used to evaluate pre/post- surgical outcome comparisons showed that significant changes were made (p<0.05).

## Results:

In this study, 100 patients were included and analyzed, with an average age of 66.4 ± 7.8 years and a larger number of women (58%). The average body mass index was 28.6 ± 3.4 kg/m^2^ and there was an average of 6.2 years of symptom duration before surgery, all reported by the patients. The improvements to patient-reported and functional outcomes were highly significant after surgery. There were significant improvements in KOOS scores across all domains. The pain domain improved from 45.6 ± 10.4 to 82.1 ± 8.9 and the quality-of-life domain improved from 34.5 ± 13.1 to 76.9 ± 8.8 (p < 0.001). There were also significant improvements in physical functioning (41.2 ± 9.4 to 80.5 ± 7.8) and mental health (57.6 ± 8.7 to 81.2 ± 7.3) according to SF-36 scores (p < 0.001). There were marked improvements in functional performance. The average timed up-and-go time decreased from 18.4 to 10.2 seconds and the average six-minute walk distance increased from 230.7 to 360.8 m (p < 0.001). Overall, the majority of patients were satisfied with the results of their surgery (68%) and 71% reported complete pain relief. There were very few complications, which included superficial infection (3%), deep venous thrombosis (2%) and stiffness (4%). Pain relief, functional performance and quality of life were strongly correlated (r = 0.81, p < 0.001), which indicates that there was substantial improvement in these areas after total knee replacement.

Table 1 (see PDF) provides the baseline characteristics of 100 patients, showing a mean age of 66.4 years, female predominance and average BMI of 28.6 kg/m^2^, typical of the osteoarthritis population. Table 2 (see PDF) demonstrates significant postoperative improvement in all KOOS domains, especially pain, daily activities and quality of life. Table 3 (see PDF) compares SF-36 scores and reveals marked enhancement in both physical and mental health components after surgery. Table 4 (see PDF) shows improved functional performance, with faster timed up-and-go results and greater six-minute walk distances. Table 5 (see PDF) provides patient satisfaction data, indicating that most participants experienced notable pain relief and high satisfaction. Table 6 (see PDF) demonstrates a strong correlation between pain relief and functional recovery, confirming that reduced pain led to better mobility. Table 7 (see PDF) compares demographic factors and shows that younger and lower BMI patients achieved better postoperative outcomes. Table 8 (see PDF) highlights a strong positive correlation between functional improvements and quality of life. Table 9 (see PDF) reports minimal postoperative complications, confirming procedural safety. Finally, Table 10 (see PDF) summarizes overall outcome gains, demonstrating significant improvement in pain, function, endurance and quality of life following total knee replacement.

## Discussion:

The information in this report shows a great improvement in pain and function and quality of life from total knee replacement surgery based on both patient-reported outcomes and functional measurements [8]. The greatly improved knee injury and osteoarthritis outcome score (KOOS) and short form 36-item health survey (SF-36) indicate that the improvements seen following total knee replacement go beyond the radiographic findings and improve the well-being of the patient as determined by the patient [9]. The improvements seen in this study are supported by more recent literature that highlights pain relief and the return to daily functional activity as two major factors impacting satisfaction following surgery [10, 11]. The results from the functional tests indicate that the patients improved their ability to get around and their endurance levels. The timed-up and go results show a decrease in an average of 45% in the amount of time it takes to perform this test and an increase of an average of 56% in the amount of distance walked in six minutes. Similar improvements have been documented in modern studies of patient rehabilitation outcomes from surgery [12, 13]. There is a strong correlation between pain relief, mobility and quality of life and these results clearly show the interdependence of recovery from surgery. The age of the patients and the patients' body mass index (BMI) is predictive of their postoperative scores being higher than those of older patients and those with a higher BMI, which is consistent with the findings in existing studies related to predicting recovery following surgery [14]. It should also be noted that even though the younger and lower BMI subgroups had the best scores, significant improvement was seen across the entire sample, demonstrating the broad effectiveness of this procedure. The high postoperative satisfaction rates and low rates of complications support the safety and clinical value of total knee replacement surgery [15]. Utilizing both patient-reported outcomes and functional testing provides a comprehensive understanding of recovery after total knee replacement procedures. This combined approach to the evaluation of recovery provides orthopaedic surgeons and patients with a more integrated assessment of postoperative recovery. Limitations of this study include the fact that it was conducted at a single institution, had a moderate sample size and had a relatively short follow-up time. Future multicenter studies with long-term follow-up periods are necessary to further assess the durability of the results from total knee replacement surgery.

## Conclusion:

Patients suffering from advanced osteoarthritis see marked improvement following total knee replacement in terms of their levels of pain, ability to function, quality of life and satisfaction after surgery. Patients who had total knee replacement surgery experienced few complications when rehabilitation was provided in a structured environment.

## References

[1] Chang J (2022). Patient Prefer Adherence..

[2] Al Thaher Y (2021). Adv Orthop..

[3] Woodland N (2023). J Clin Med..

[4] Trieu J (2020). J Clin Med..

[5] Mukartihal RK (2022). Int Orthop..

[6] Rodriguez-Merchan EC (2021). Arch Bone Jt Surg..

[7] Klem NR (2020). Osteoarthr Cartil Open..

[8] Singh M (2025). J Arthroplasty..

[9] Ayers DC (2023). J Arthroplasty..

[10] Alomran AS (2022). Ann Afr Med..

[11] Althomali OW (2025). J Clin Med..

[12] Liu Q (2022). Lancet Rheumatol..

[13] Baghbani-Naghadehi F (2022). BMC Musculoskelet Disord..

[14] Wu KA (2025). J Arthroplasty..

[15] Hill BG (2023). J Arthroplasty..

